# Pediatric antibody responses to SARS-CoV-2 after infection and vaccination in Calgary, Canada

**DOI:** 10.1186/s12879-024-09615-3

**Published:** 2024-07-18

**Authors:** Leah J. Ricketson, Emily J. Doucette, Isabella Alatorre, Tarannum Tarannum, Joslyn Gray, William Booth, Graham Tipples, Carmen Charlton, Jamil N. Kanji, Kevin Fonseca, James D. Kellner

**Affiliations:** 1 Department of Pediatrics, Cumming School of Medicine, University of Calgary, Calgary, AB Canada; 2 Public Health Laboratory, Alberta Precision Laboratories, Calgary, AB Canada; 3 Li Ka Shing Institute of Virology, University of Alberta, Edmonton, AB Canada; 4 Department of Medical Microbiology and Immunology, Faculty of Medicine and Dentistry, University of Alberta, Edmonton, AB Canada; 5 Department of Pathology and Laboratory Medicine, Cumming School of Medicine, University of Calgary, Calgary, AB Canada; 6 Department of Laboratory Medicine and Pathology, Faculty of Medicine and Dentistry, University of Alberta, Edmonton, AB Canada; 7 Division of Infectious Diseases, Department of Medicine, Cumming School of Medicine, University of Calgary, Calgary, AB Canada; 8 Department of Microbiology, Immunology & Infectious Diseases, University of Calgary, Calgary, AB Canada; 9 Alberta Children’s Hospital Research Institute, University of Calgary, Calgary, AB Canada

**Keywords:** COVID-19, Pediatrics, SARS-CoV-2, SARS-CoV-2 immunity, Hybrid immunity

## Abstract

**Background:**

There are few reports of longitudinal serologic responses in children following Sars-CoV-2 infection and vaccination. This study describes longitudinal SARS-CoV-2 antibody responses following infection, vaccination, or both (hybrid immunity) in a cohort of Canadian children. The objectives of our study were to compare antibody levels following SARS-CoV-2 infection, vaccination, and hybrid immunity and to examine antibody decline after final antigen exposure.

**Methods:**

The Alberta Childhood COVID-19 Cohort (AB3C) study was a prospective longitudinal cohort study conducted from July 2020 to September 2022 with repeat sampling across 5 visits. Children under 18 years of age were enrolled for serial measurement of antibody responses to SARS-CoV-2 virus vaccine and infection.

**Results:**

The final sample size was 919; participants were 50.5% female, 48.2% were > 12 years and 88.5% were white ethnicity. The median peak spike IgG level of those with only infection was not different from those with no vaccination or infection (233 AU/mL (IQR: 99–944 AU/mL) vs. 3 AU/mL (IQR: 1–5 AU/mL; *P* = 0.1765). Participants with infections after vaccination had higher IgG levels than those where infection preceded vaccination (median: 36,660 (IQR: 22,084 − 40,000 AU/mL) vs. 17,461 AU/mL (IQR: 10,617 − 33,212 AU/mL); *P* < 0.0001). In a linear mixed methods model, children with infection-only had low levels of antibody that stayed stable over the study duration without further antigen exposures. Those with infection after vaccination had the slowest rate of antibody decline over time at 4% (95%CI: 2-5%) per week, compared with children where infection preceded vaccine 7% (95%CI: 6-8%) per week.

**Conclusions:**

Children with hybrid immunity conferred through vaccination (2 + doses) followed by a SARS-CoV-2 infection had the highest and longest lasting antibody levels, compared to children who had an infection followed by vaccination, vaccination-only, or infection-only. The longer-term clinical importance of these findings, related to prevention of repeated infections and severe outcomes and need for further vaccine doses, is not yet known.

**Supplementary Information:**

The online version contains supplementary material available at 10.1186/s12879-024-09615-3.

## Background

The World Health Organization declared the severe acute respiratory syndrome coronavirus 2 (SARS-CoV-2) pandemic on March 11, 2020, which was followed by the rapid development and uptake of vaccines to protect against COVID-19 infections. The evolution of numerous variants of the original virus and very high levels of acquired infection, with or without vaccination, have made it increasingly complex to evaluate immune responses in persons with multiple exposures.

The main goal of vaccination against COVID-19 is the development of neutralising antibodies (nAbs) against the receptor binding domain of the spike protein in recipients [1]. These nAbs are strongly correlated with protection against severe COVID-19 [2, 3]. However, nAbs have been shown to wane over time, which may lead to reduced effectiveness in preventing infection and severe disease. This highlights the rationale for booster doses especially for high-risk populations [4]. Both infection and vaccination increase nAbs against the spike protein. The combination of immune responses to both infection and vaccination, often called “hybrid immunity,” results in higher levels and more gradual decay of antibodies over time compared to vaccine-only or infection-only induced immunity [5–16]. Hybrid immunity has been demonstrated to provide better protection against symptomatic and severe SARS-CoV-2 infection compared to infection-only and vaccine-only induced immunity [17–19].

Before the Omicron variant emerged, reported symptomatic infections were less common in children than in adults [20]; however, children more frequently have asymptomatic infections and milder disease [21–26]. Most studies examining seropositivity following vaccination and infection have focused on adults [9, 27–31]. Few studies have evaluated immune responses in cohorts of children on a longitudinal basis following vaccination, infection, and combinations of both, though some have looked at levels of antibodies in children mostly after acquired infection [32–37].

We conducted a longitudinal study of SARS-CoV-2 antibody levels in children over a two-year period during the COVID-19 pandemic in Calgary, Canada. During this time children experienced many different combinations of exposure to SARS-CoV-2 antigens through infection, vaccination, or both. We also examined the duration of antibody levels in children following their last antigen exposure.

## Methods

### Study recruitment

The Alberta Childhood COVID-19 Cohort (AB3C) enrolled 1035 children to attend up to five study visits between July 2020 and September 2022 for blood collection to measure nucleocapsid and spike IgG antibodies. Children with a previous diagnosis of COVID-19 were identified by Alberta Health Services (AHS) and invited to participate. Infection-naive children were recruited through a social media announcement. All participants were under 18 years of age at enrollment. The study received approval from the University of Calgary Conjoint Health Research Ethics Board (Ethics ID: REB20-0480). Detailed methods have been reported previously [38, 39].

At each study visit, parents, guardians, or participants (older children) completed an online survey to report on health history, demographic features, and vaccination with mRNA vaccines, with vaccinations later confirmed through the AHS vaccine registry.

Across visits there was variability in how many children completed blood draws due to missed visits and withdrawals. Participants who did not provide consent to access vaccination records and those whose self-reported vaccinations were discordant with the AHS records of vaccination were excluded as there was potential for misclassification. Results from participants who withdrew part way through the study were included up to the time of withdrawal.

### Laboratory

Venous blood was collected and sent to the Alberta Precision Laboratories, Public Health Laboratory for processing. Nucleocapsid and spike IgG levels were determined using the Abbott Alinity chemiluminescent microparticle immunoassays to detect binding antibodies either to the nucleocapsid protein or spike protein receptor binding domain (RBD) of SARS-CoV-2 spike protein’s S1 subunit [40, 41]. The Abbott AdviseDx Architect SARS-CoV-2 IgG II immunoassay detects IgG against the spike RBD with a positive cut off of > 50 arbitrary units per millilitre (AU/mL) [40]. The AdviseDx IgG II test has high sensitivity and specificity for detecting IgG against the RBD [42]. This immunoassay has an upper limit of 40,000 AU/mL, therefore this is the maximum reported level of IgG for this study [40]. The Abbott SARS-CoV-2 IgG (or CoV-2 IgG) detects IgG against the nucleocapsid protein [41]. Nucleocapsid antibodies are reported as signal to cut off ratio (S/C) and samples with a value of 1.4 and greater were considered positive [41].

### Definitions

Infection status was based on self-report of a laboratory conducted positive polymerase chain reaction (PCR) or rapid antigen test (RAT) test prior to or throughout the study. PCR results were confirmed through AHS records, but RAT results were based only on self-report. Those who reported an exposure and had serology results to suggest infection were also considered to have been infected. A newly positive nucleocapsid antibody test, and/or a newly positive spike IgG in an unvaccinated child, were assumed to indicate a new infection regardless of whether the participant was aware they had been infected.

All participants were included in one of 8 immune status groups defined by their SARS-CoV-2 infection and vaccination status. These included: no infection or vaccination, infection-only, vaccine only-1 dose, infection and 1 dose of vaccine, and vaccine only with 2 or more doses. There were also 3 hybrid immunity groups: infection followed by 2 vaccine doses (IVV), 2 or more vaccine doses followed by infection (VVI), and 1 vaccine dose followed by an infection and then another vaccine dose (VIV). Some children had additional exposures. Additional File 1 describes all the exposure profiles and how they were classified for the hybrid immunity groups.

### Descriptive analysis

Demographic and clinical factors were tabulated as of the final visit for each participant including age, sex at birth, ethnicity, Indigenous status, comorbidities (asthma, immunosuppressive disorders, and other health conditions), SARS-CoV-2 infection status, vaccination status, and hybrid immunity status.

Peak spike IgG values were determined and children were classified into the immune group that reflects their immune status at the time of the peak spike measurement. The median spike IgG value of each immune status group was compared to all others using a Kruskal-Wallis test for ranked data with an alpha level < 0.05 considered significant. Dunn’s multiple comparison test was performed on each pair of groups to determine where significant differences occurred. Analysis was conducted using Prism 10 and STATA/SE version 18.0.

### Analysis of antibody durability: Linear mixed effects Model

Last antigen exposure was defined as the date of the last confirmed SARS-CoV-2 infection or vaccine dose. The time since last antigen exposure was calculated in weeks. We compared the decline of spike IgG between the main immune response groups: infection-only, vaccine-only, IVV, and VVI. Throughout the study period, the immune status group of a participant could change, so each child could provide data to different immune status groups sequentially (e.g., vaccine only initially and then VVI at later visits). Data for the mixed-effects model was restricted to only include spike IgG values where sampling occurred when a participant was within the four main immune status groups (infection-only, vaccine-only, VVI, and IVV).

Data were excluded from children whose last antigen exposure was a new infection detected by newly positive nucleocapsid IgG or a newly positive spike IgG in an unvaccinated child. For these children the date of last antigen exposure could not be determined as infection was detected at blood draw. The analysis excluded children with no infection or vaccine (no antigen exposure), infection and 1 vaccine, and 1 vaccine only as well as the VIV group as these groups were too small to consider. Nucleocapsid-only infections that were followed by a vaccine were still included as the last exposure was the vaccine. Spike IgG values following reinfections or following a third dose of vaccine were also excluded as we wanted to look at decline over time since last exposure without other exposures increasing IgG levels.

A mixed-effects linear regression model was developed to examine change in trend for log spike antibody levels (AU/mL) since the last antigen exposure. The spike values were log transformed using a natural logarithm to improve normality of residuals and allow for simpler interpretation of slopes. The model allowed for both individual trend over time as well as an average trend over time. Models were adjusted for age group and sex at birth. Age was treated as a time-varying covariate, with individuals potentially moving between the following categories over time: <5 years, 5–12 years, and 12 + years. Sex at birth was included as a time constant covariate. Infection-only was the reference group for the model. A restricted model to compare VVI and IVV directly was also developed. The model was truncated at 40 weeks due to no data past 40 weeks for the VVI group.

## Results

### Population

**Fig. 1:**
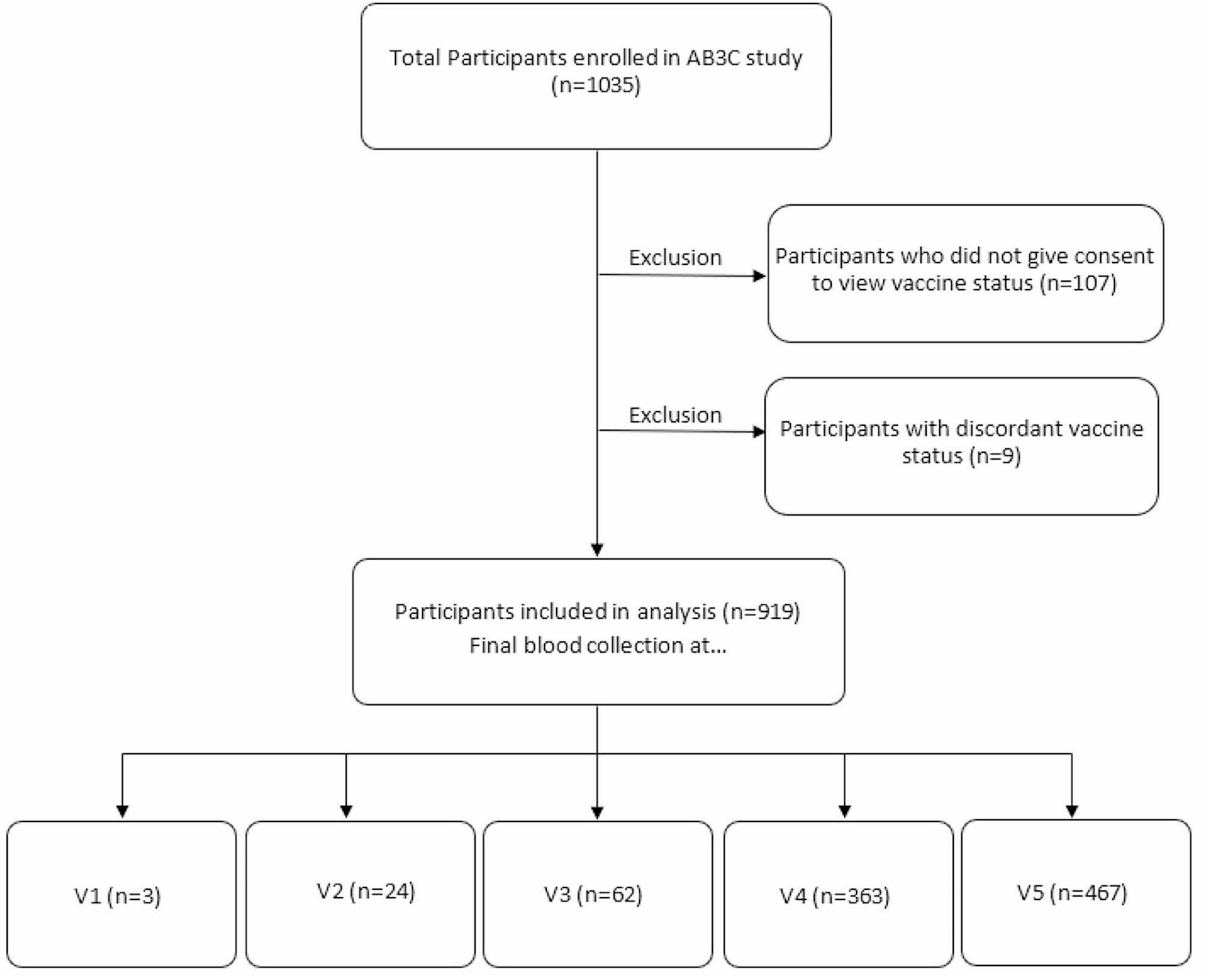
Study flow chart

There were 1035 children recruited into the AB3C study. We excluded children who did not consent to access vaccination records (*n* = 107) and children whose self-reported vaccinations were discordant with the AHS registry (*n* = 9) (Fig. 1). The final study population included 919 children. The number whose final blood draw was obtained during each of the 5 visit periods is noted in Fig. 1. The participants were 50.5% female, 48.2% were 12 years or older and 88.5% were white ethnicity (Table 1).

**Table 1:** Demographics and clinical characteristics of population

	*N* (%) (total = 919)
**Age at last blood draw**	
Under 5 years 5–11 years 12 + years	95 (10.3) 382 (41.6) 442 (48.1)
**Sex at birth**	
Female Male	464 (50.5) 455 (49.5)
**Ethnicity**	
White Other^1^ Prefer not to answer	814 (88.6) 89 (9.7) 16 (1.7)
**Indigenous status**	
Indigenous Non-Indigenous	39 (4.2) 880 (95.8)
**Health conditions**	
Asthma Immunosuppressed Other^2^ None	103 (11.2) 10 (1.1) 622 (67.7) 184 (20.0)

1. Other ethnicities include Asian, Black, and mixed-race.

2. Other health conditions include chronic neurological disease, celiac disease, ADHD, blood disorders, cardiovascular disease, diabetes, cancer, and more.

### Infections and vaccinations

Throughout the study 563/919 children (61.3%) had a single SARS-CoV-2 infection; 75 (8.2%) had 2 infections, and 1 (0.15%) had 3 infections, for a total of 639 (69.5%) with 1 or more infections. Most first infections (398/563; 70.7%) occurred during the Omicron variant period after visit 3.

Although most infections were determined by positive PCR or RAT as well as serology indicating infection, 163 infections were determined by increase in nucleocapsid antibody at blood draw and 23 were based on an increase in spike antibody with no history of vaccination. None of these children reported symptomatic illnesses prior to serology testing indicating 186/639 infections (29.1%) were asymptomatic.

There was 1 hospital admission and 3 emergency department visits reported that were related to COVID-19 infections across all 5 study visits [38]. The proportion of children with spike IgG rose from 8.8% (94/908) tested at V1 to 94.6% (442/467) tested at V5 after infection, vaccination, or both. By study end, 85.5% (786/919) of all participants had received at least one dose of vaccine, and 79% (728/919) received 2 or more doses.

The proportion of children who received 2 or more doses of vaccine was 5/100 (5%) for children under 5 yearss, 262/404 (65%) for children 5–11 years, and 390/415 (94%) for children 12 + years.

### Peak antibody levels by Immune Response groups

**Fig. 2:**
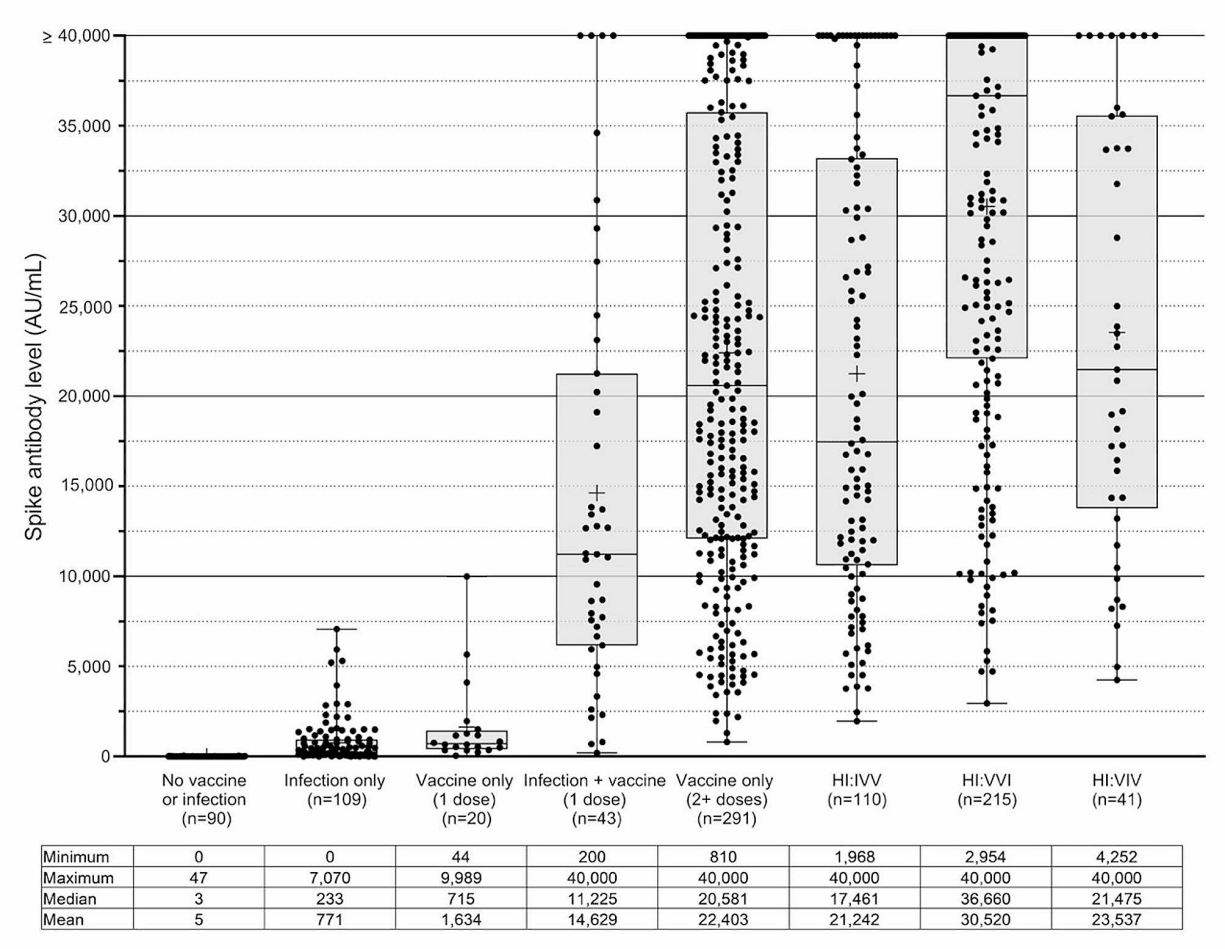
Peak spike antibody levels in 8 immune response groups. The group ranges, mean and median are shown below the boxplot. The group mean is represented by plus sign (+) while median is shown by horizontal line within boxplot

The mean, median, and range of peak spike antibody levels are shown for each of 8 immune status groups in Fig. 2. The median peak spike IgG level of those with infection-only, did not significantly differ from those without a history of vaccination or infection (233 AU/mL (IQR: 99–944 AU/mL) vs. 3 AU/mL (IQR: 1–5 AU/mL) (*P* = 0.1765)). The median IgG level of participants who had received 2 + doses of vaccine (20,581 AU/mL; IQR: 12,092 − 35,753 AU/mL) was significantly higher than those with infection-only (*P* < 0.0001), but significantly lower than those with hybrid immunity grouping VVI (36,660 AU/mL; IQR: 22,084 − 40,000 AU/mL) (*P* < 0.0001). There was no difference between those with 2 + vaccine doses and those with hybrid immunity group IVV (17,461 AU/mL; IQR: 10,617 − 33,212 AU/mL) (*P* > 0.9999). Participants with breakthrough hybrid immunity (VVI) had significantly higher median IgG levels than those with hybrid immunity where infection preceded vaccination (IVV) (*P* < 0.0001). Additional file 2 shows comparisons between the peak IgG levels for all immune groups.

### Mixed-effects model

**Fig. 3:**
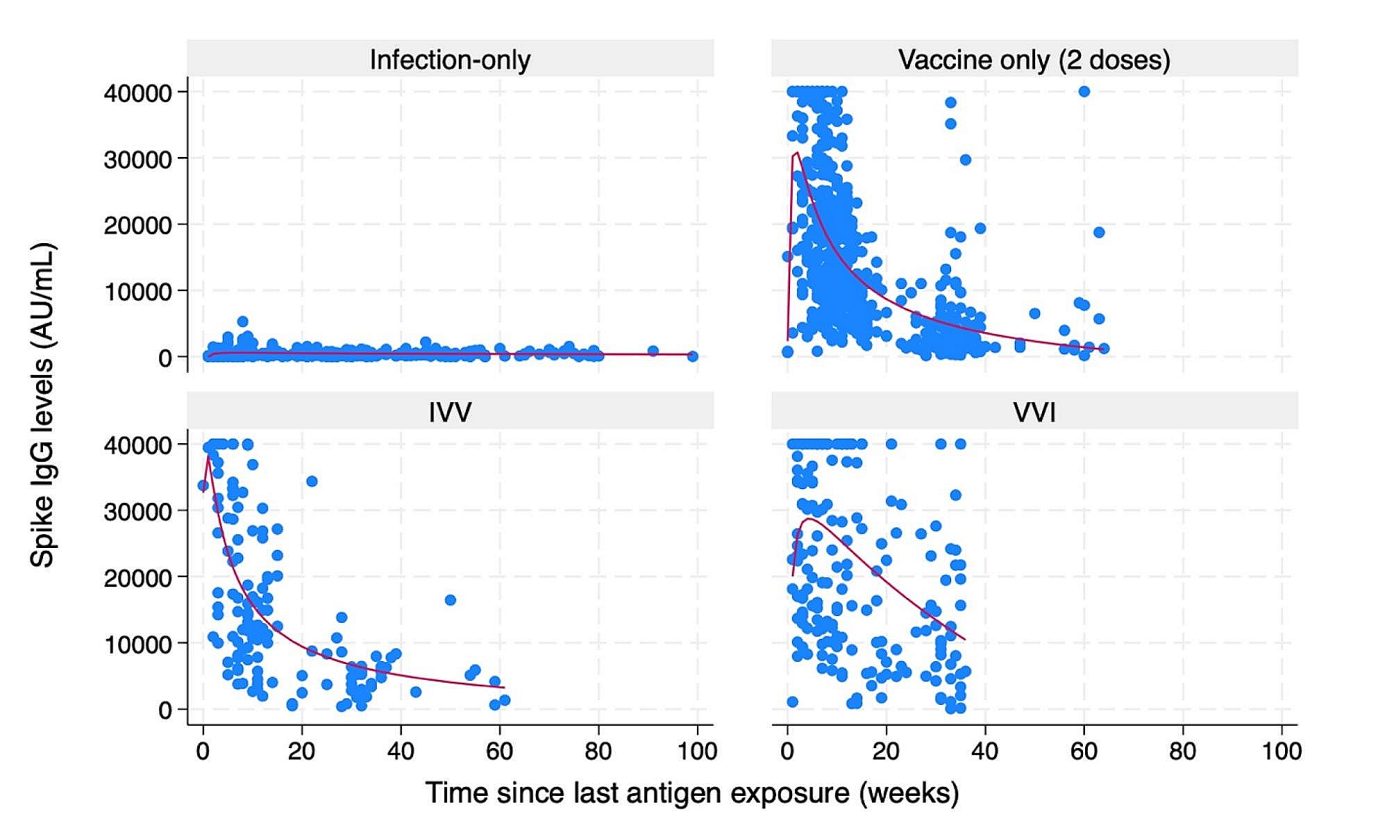
Scatterplot of spike IgG values (AU/mL) with polynomial curve before log transformation vs. time in weeks since last antigen exposure

**Fig. 4:**
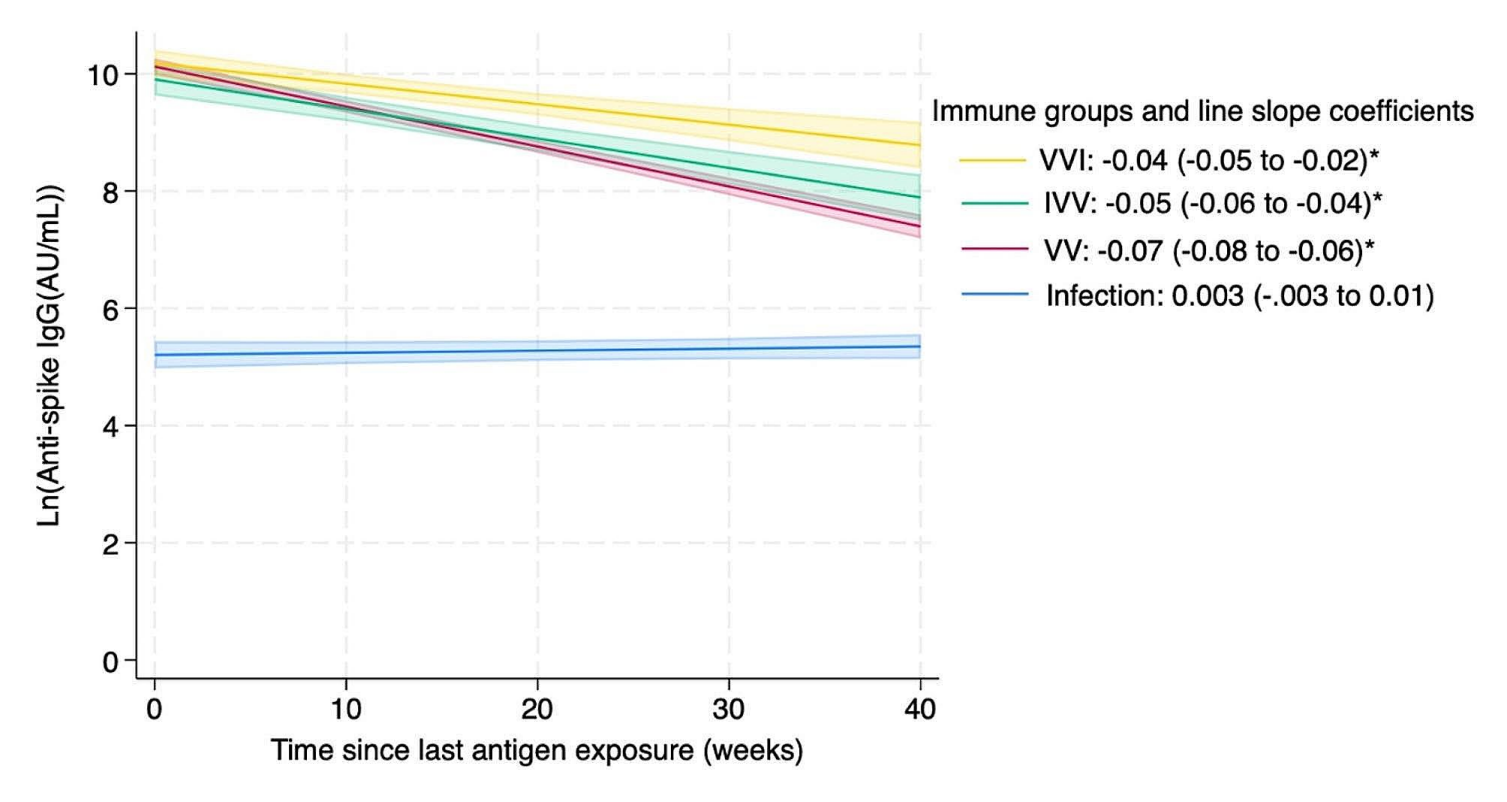
Mixed-effects model showing duration of log antibody levels over time in weeks from last infection/vaccine exposure (*N* = 658 children, 1283 spike IgG measurements). The slope coefficients are indicated in the legend. The model was also adjusted for age and sex. * indicates a slope that is significantly different from zero. All slopes except infection-only were significant with *P* < 0.001

We excluded 180 children who were never exposed to antigens, received only one vaccine by study conclusion, were in the hybrid immunity VIV group, or did not have any spike values to contribute. We also excluded 81 cases as their last antigen exposures was indicated only by an increase in nucleocapsid or spike levels and so the infection date was not known. The final sample analyzed included 658 children, with 1283 spike IgG data points, including 1 to 5 data points per child (Figs. 3 and 4).

The infection-only group had lower levels of IgG and the level declined by 0.3% per week which was not significant (95%CI: -0.3-1%) (Fig. 4). IgG levels for vaccine-only (2 doses) started much higher than infection-only but declined over time by approximately 7% (95%CI: 6-8%) per week, IVV declined by 5% (95%CI: 4–6%) per week and VVI IgG levels declined at approximately 4% (95%CI: 2-5%) per week. With the model restricted to compare IVV and VVI, spike IgG for the VVI group declined at 4% (95%CI: 2-5%) per week, which was significantly slower than the IVV group at 7% (95%CI: 6-8%) per week. Although all groups except infection-only waned after the last antigen exposure, the IgG levels still remained well above the positivity threshold even up to 40 weeks post exposure. The model was adjusted for age and sex with the overall model including all children of all ages combined. Age-group specific analysis within the different immunity groups was not performed since the number of data points from children under 5 years with 2 or more doses of vaccine was very small (5/100, 5%).

## Discussion

This cohort of Canadian children and adolescents was followed longitudinally through the peak of the COVID-19 pandemic (summer 2020 to autumn 2022). At study conclusion, the participants were highly vaccinated and frequently infected mainly with mild or asymptomatic infections. The children demonstrated high levels of binding antibody, particularly after vaccination with or without infection (hybrid immunity). Those with hybrid immunity due to breakthrough infection after vaccination with at least 2 doses of mRNA vaccine (VVI) had the highest and longest lasting antibody levels, compared to children who had an infection followed by vaccination (IVV), vaccination-only, or infection-only. Numerous adult studies have focused on studying antibody responses towards COVID-19, but there have been fewer studies examining antibody response in children [43–46].

Hybrid immunity has been demonstrated to result in greater antibody response than vaccine or infection alone [16, 44, 47]. The order of these immunity generating events may also be relevant in the development of optimal immune response. Similar to our findings, adult studies found infection after vaccination had the highest median antibody levels, higher than the groups with vaccination after infection, as well as the vaccine-only group and infection-only group [16, 48]. In children, vaccine after infection also confers high levels of antibodies [49, 50].

In children, two or more mRNA vaccine doses led to high median IgG levels, as did vaccination preceded (IVV) or followed (VVI) by infection. The highest peak IgG response was in the VVI group and antibody decline over time was slowest in this group. Yamamoto et al. also found hybrid immunity to have the slowest waning of antibody levels over time but did not distinguish between infection prior to or following vaccination [51]. They demonstrated that 3 doses of vaccine waned more slowly than 2 doses, which was something we were unable to assess due to a limited number of measurements that occurred after a third vaccine dose [51]. Waning antibody levels are expected with time [8, 44, 52]. We observed considerable diversity in antibody waning to the end of the current study period, with some children maintaining spike IgG levels above 20,000 AU/mL up to 40 weeks after their last antigen exposure with no documented subsequent exposures. Further, we found antibody levels did not wane significantly in those with infection-only, although the levels were much lower to start. Another study also showed no significant waning of naturally acquired antibodies over 6–8 months in children [35]. We found spike IgG remained well above the positivity threshold even at 40 weeks after last antigen exposure which others have also found in adults [53] and children [37].

Measurement of serum IgG binding antibodies provides important information about the immune response to SARS-CoV-2 vaccines and infections but does not provide information about other humoral or the cell-mediated responses. One study suggested that children may mount a more robust innate immune response (as evidenced by higher levels of inflammatory cytokines) than adults and lower levels of neutralizing antibody after infection [54]. In addition, while our study described much higher antibody levels in vaccine recipients than those with infection only, it is unclear what additional protective benefit may have been provided. In this study of generally healthy children, there were very few SARS-CoV-2 infections that required emergency department visits or hospital admissions. Infection-only immunity may result in lower antibody levels but, with concurrent cell-mediated immunity, may still provide adequate protection against severe outcomes. A meta-analysis by Bobrovitz et al. showed that infection-only immunity provided good protection against hospitalization and severe disease but lower protection against reinfection and the protection waned over time [19]. Several studies have shown that hybrid immunity had the strongest and most durable protection against reinfection, severe disease, and hospitalization in adults [13, 17, 19], and reinfection in children [55].

We found 29% of children in this study had serology results indicating a recent infection without a symptomatic illness and without a positive RAT or PCR test. This may have been an underestimate of the frequency of asymptomatic infections as other children had nucleocapsid antibody increases that did not reach the threshold for positivity but were suggestive of infection. Two other studies reported approximately 44% of asymptomatic infections in children [33, 56]. Other studies have shown seropositivity to be similar in children and adults but with fewer symptoms in children, suggesting mild and asymptomatic infections in children often go undetected [21, 36, 57].

This study had some limitations. We examined binding antibody levels only, not neutralizing antibody levels or cellular immunity. However, binding antibody and neutralizing antibody measurements are highly correlated, and both can be used as correlates of protection against future infection and severe disease [58–61].

We previously reported that the AB3C study enrolled limited numbers of participants from lower income families; however, in our previous analysis income was not a significant factor in choice to vaccinate when other factors were considered [39]. Similarly, visible minorities and Indigenous groups were not fully represented in the study. In Alberta, the 2021 national census found that 6.8% of the population identified as Indigenous people. Within the study population 4.2% identified as Indigenous [62]. In the 2016 national census 33.7% of the population of Calgary identified as a visible minority with the most common being South Asian and Chinese [63]. Within the study population, 9.7% identified as a visible minority. Therefore, these results may not be completely generalizable to visible minorities or lower income children.

Due to the staggered age-specific rollout of COVID-19 vaccines in Canada, starting with adolescents and later including younger age groups, we could not conduct age group-specific examination of the effect of age on antibody levels. Nearly all children aged 12 + years were vaccinated with 2 or more doses of vaccine, whereas nearly all children under 5 years received no vaccine doses. For children 5–11 years, 65% received 2 or more vaccine doses. However, age (and sex) were included as covariates in the mixed effects model which provided results for all children combined. This enabled analysis of the effects of both vaccination and infection on antibody levels.

The time span of this study included the periods of multiple SARS-CoV-2 variants although most infections occurred later in the study during the Omicron variant period. Those first infected prior to vaccination most often had ancestral or Alpha variant infections and those first infected after vaccinations most often had Omicron variant infections. We cannot distinguish whether the difference in humoral response before or after vaccination is related to the variants causing infection, as well as the order of antigen exposures.

Children in this observational study had a multitude of exposures to SARS-CoV-2 infections and vaccines. Restricting the groups to comparable combinations of infection and/or vaccines within each group reduced the number of observations, but ensured each group was comprised of children with uniform exposures.

## Conclusions

This longitudinal cohort of children followed through the COVID-19 pandemic were highly vaccinated and frequently infected, and demonstrated high levels of binding antibody, particularly after vaccination with or without infection. Those with hybrid immunity conferred through vaccination with at least 2 doses of mRNA vaccines followed by a SARS-CoV-2 infection had the highest and longest lasting antibody levels, compared to children who had an infection followed by vaccination, vaccination-only, or infection-only. The longer-term clinical relevance of these findings, related to prevention of repeated infections and severe outcomes, is not yet known.

### Electronic supplementary material

Below is the link to the electronic supplementary material.

Additional file 1: Hybrid immune response breakdown per group at each participant’s visit with their peak spike value

Additional file 2: Comparison of median peak IgG levels between different immune response groups using Kruskal-Wallis Test with Dunn’s Multiple Comparison test

## Data Availability

The datasets used and/or analyzed during the current study are available from the corresponding author on reasonable request.
