# Supplementary material for: Pediatric antibody responses to SARS-CoV-2 after infection and vaccination in Calgary, Canada
Source: BMC Infect Dis. 2024 Jul 18;24:705. doi: 10.1186/s12879-024-09615-3 (PMC11256562; doi:10.1186/s12879-024-09615-3)
Supplement: Supplementary file 1 — Additional file 1: Hybrid immune response breakdown per group at each participant’s visit with their peak spike value [file 12879_2024_9615_MOESM1_ESM.docx]

**Additional File 1.** Hybrid immune response breakdown per group at each participant’s visit with their peak spike value.

| **Hybrid Immune Response Group** | **N (total=366)** | **Total Antigen Exposures^a^** | **Median Maximum Spike Value (AU/mL)** |
| --- | --- | --- | --- |
| IVV | **110** | | |
|  | 82 | IVV | 16445 |
|  | 8 | IVVI | 15865 |
|  | 9 | IVVV | 17561 |
|  | 2 | IVVIV | 13110 |
|  | 3 | IIVV | 17848 |
|  | 3 | IVVVI | 20649 |
|  | 2 | IVIV | 16445 |
|  | 1 | IIVVI | 40000 |
| VIV | **41** | | |
|  | 37 | VIV | 16607 |
|  | 3 | VIVI | 14354 |
|  | 1 | VIVV | 17222 |
| VVI | **215** | | |
|  | 160 | VVI | 16550 |
|  | 31 | VVIV | 17693 |
|  | 20 | VVVI | 15923 |
|  | 2 | VVII | 14875 |
|  | 2 | VVIVI | 18031 |
| 1. “I” indicates an infection and “V” indicates vaccination. | | | |
