# Supplementary material for: Pediatric antibody responses to SARS-CoV-2 after infection and vaccination in Calgary, Canada
Source: BMC Infect Dis. 2024 Jul 18;24:705. doi: 10.1186/s12879-024-09615-3 (PMC11256562; doi:10.1186/s12879-024-09615-3)
Supplement: Supplementary file 2 — Additional file 2: Comparison of median peak IgG levels between different immune response groups using Kruskal-Wallis Test with Dunn’s Multiple Comparison test [file 12879_2024_9615_MOESM2_ESM.docx]

**Additional File 2:** Comparison of median peak IgG levels between different immune response groups using Kruskal-Wallis Test with Dunn’s Multiple Comparison test.

| **Group 1** | | | **Group 2** | | |  |
| --- | --- | --- | --- | --- | --- | --- |
| **Immune Response Group** | **Median IgG (AU/mL)** | **IQR (AU/mL)** | **Immune Response Group** | **Median IgG (AU/mL)** | **IQR (AU/mL)** | **Adjusted P-Value** |
| No vaccine or infection | 3 | 1-5 | Infection only | 233 | 99-944 | 0.1805 |
| No vaccine or infection | 3 | 1-5 | Vaccine only (1 dose) | 715 | 400-1,446 | 0.9062 |
| No vaccine or infection | 3 | 1-5 | Infection + vaccine (1 dose) | 11,225 | 6,162-21,260 | <0.0001 |
| No vaccine or infection | 3 | 1-5 | Vaccine only (2+ doses) | 20,581 | 12,092-35,753 | <0.0001 |
| No vaccine or infection | 3 | 1-5 | HI:IVV | 17,461 | 10,617-33,212 | <0.0001 |
| No vaccine or infection | 3 | 1-5 | HI:VVI | 36,660 | 22,084-40,000 | <0.0001 |
| No vaccine or infection | 3 | 1-5 | HI:VIV | 21,475 | 13,779-35,570 | <0.0001 |
| Infection only | 233 | 99-944 | Vaccine only (1 dose) | 715 | 400-1,446 | >0.9999 |
| Infection only | 233 | 99-944 | Infection + vaccine (1 dose) | 11,225 | 6,162-21,260 | <0.0001 |
| Infection only | 233 | 99-944 | Vaccine only (2+ doses) | 20,581 | 12,092-35,753 | <0.0001 |
| Infection only | 233 | 99-944 | HI:IVV | 17,461 | 10,617-33,212 | <0.0001 |
| Infection only | 233 | 99-944 | HI:VVI | 36,660 | 22,084-40,000 | <0.0001 |
| Infection only | 233 | 99-944 | HI:VIV | 21,475 | 13,779-35,570 | <0.0001 |
| Vaccine only (1 dose) | 715 | 400-1,446 | Infection + vaccine (1 dose) | 11,225 | 6,162-21,260 | 0.0455 |
| Vaccine only (1 dose) | 715 | 400-1,446 | Vaccine only (2+ doses) | 20,581 | 12,092-35,753 | <0.0001 |
| Vaccine only (1 dose) | 715 | 400-1,446 | HI:IVV | 17,461 | 10,617-33,212 | <0.0001 |
| Vaccine only (1 dose) | 715 | 400-1,446 | HI:VVI | 36,660 | 22,084-40,000 | <0.0001 |
| Vaccine only (1 dose) | 715 | 400-1,446 | HI:VIV | 21,475 | 13,779-35,570 | <0.0001 |
| Infection + vaccine (1 dose) | 11,225 | 6,162-21,260 | Vaccine only (2+ doses) | 20,581 | 12,092-35,753 | 0.1302 |
| Infection + vaccine (1 dose) | 11,225 | 6,162-21,260 | HI:IVV | 17,461 | 10,617-33,212 | 0.7767 |
| Infection + vaccine (1 dose) | 11,225 | 6,162-21,260 | HI:VVI | 36,660 | 22,084-40,000 | <0.0001 |
| Infection + vaccine (1 dose) | 11,225 | 6,162-21,260 | HI:VIV | 21,475 | 13,779-35,570 | 0.3985 |
| Vaccine only (2+ doses) | 20,581 | 12,092-35,753 | HI:IVV | 17,461 | 10,617-33,212 | >0.9999 |
| Vaccine only (2+ doses) | 20,581 | 12,092-35,753 | HI:VVI | 36,660 | 22,084-40,000 | <0.0001 |
| Vaccine only (2+ doses) | 20,581 | 12,092-35,753 | HI:VIV | 21,475 | 13,779-35,570 | >0.9999 |
| HI:IVV | 17,461 | 10,617-33,212 | HI:VVI | 36,660 | 22,084-40,000 | <0.0001 |
| HI:IVV | 17,461 | 10,617-33,212 | HI:VIV | 21,475 | 13,779-35,570 | >0.9999 |
| HI:VVI | 36,660 | 22,084-40,000 | HI:VIV | 21,475 | 13,779-35,570 | 0.3262 |
